# Comparison of Dental Surface Image Registration and Fiducial Marker Registration: An In Vivo Accuracy Study of Static Computer-Assisted Implant Surgery

**DOI:** 10.3390/jcm10184183

**Published:** 2021-09-16

**Authors:** Yen-Ting Han, Wei-Chun Lin, Fang-Yu Fan, Chih-Long Chen, Chia-Cheng Lin, Hsin-Chung Cheng

**Affiliations:** 1 Department of Dentistry, Shin Kong Wu Ho-Su Memorial Hospital, Taipei 111, Taiwan; M011662@ms.skh.org.tw (Y.-T.H.); jlonge.tw@yahoo.com.tw (C.-L.C.); 2 School of Dental Technology, College of Oral Medicine, Taipei Medical University, Taipei 110, Taiwan; weichun1253@tmu.edu.tw (W.-C.L.); fish884027@tmu.edu.tw (F.-Y.F.); 3 School of Dentistry, College of Oral Medicine, Taipei Medical University, Taipei 110, Taiwan; g4808@tmu.edu.tw; 4 Department of Dentistry, Taipei Medical University Hospital, Taipei 110, Taiwan

**Keywords:** computer-assisted implant surgery, digital flow in dental surgery, accuracy, CT model registration, clinical research

## Abstract

This study compared the accuracy of static computer-assisted implant surgery (sCAIS) planned through dental surface image registration and fiducial marker registration. Stone models of 30 patients were converted into digital dental casts by using a desktop scanner. Cone-beam computed tomography (CBCT) was performed and superimposed to the digital dental casts with two methods: matching the dental surface images or matching the fiducial markers on a stereolithographic radiographic template. Following the implant planning, stereolithographic surgical guides were fabricated, and 56 fully guided implants were inserted by the same doctor. Deviations between planned and inserted implants were measured and compared using postoperative CBCT images. After adjustment for other potential influencing factors, compared with the fiducial marker registration group, significantly larger mean lateral deviations were noted in the dental surface registration group at both the implant platform and apex (*p* = 0.0188 and 0.0371, respectively). However, the mean lateral deviations for the dental surface registration (0.83 ± 0.51 mm at implant platform and 1.24 ± 0.68 mm at implant apex) were comparable to the literature. In conclusion, our findings indicate that although sCAIS planned using dental surface image registration was not statistically as accurate as that using fiducial marker registration, its accuracy was satisfactory for clinical use.

## 1. Introduction

Dental implants have been extensively used for both functional and esthetic rehabilitation in dentistry, and a correct three-dimensional (3D) implant position is essential for an optimal esthetic outcome and long-term functional success of implant reconstruction [1]. To achieve this goal, an appropriate implant position and angulation in relation to the surrounding bone and remaining teeth is critical. Inaccurate implant positioning may not only involve esthetic impairments of implant restorations but may also result in complications related to implant failure [2]. Although the implant position can be estimated by standardizing the two-dimensional intraoral or panoramic radiographs [3,4], cone-beam computed tomography (CBCT) allows 3D visualization of dentoalveolar structures at a lower radiation dosage than with helical computed tomography (CT); CBCT has popularized computer-assisted or computer-guided implant surgery for the last two decades [5]. Computer-assisted implant surgery allows optimal prosthetic and surgical treatment planning and accurate implant placement according to the patient’s anatomic condition [6]. It also helps in identifying anatomically sensitive structures and avoiding surgical complications such as sinus perforation and mandibular nerve injury [5].

Two computer-assisted implant surgery systems have been developed: dynamic navigation and static computer-assisted implant surgery (sCAIS). sCAIS is a system that transfers the predetermined virtual implant position to the surgical site through a physical surgical guide (template) [7]. On the basis of the virtual treatment plan, computer-aided design and computer-aided manufacturing (CAD/CAM) procedures such as additive printing or subtractive milling are normally used for fabricating surgical guides, and stereolithography (SLA) rapid prototyping is currently the most widely used technique for making the CAD/CAM surgical guide [5]. Compared with dynamic navigation, sCAIS has easier intraoperative handling of the surgical templates, requires no additional equipment, and requires less time for the presurgical set-up and intraoperative application. The high purchase and maintenance costs of the dynamic navigation systems must be considered. Indeed, dental implantology currently seems to favor sCAIS [7].

In the planning procedure of the sCAIS, the optimal prosthetic design and bony anatomic information must be considered simultaneously. Conventionally, a special scan prosthesis with radiopaque teeth or fiducial markers is necessary to achieve this requirement [8]. When the patient wears the scan prosthesis to take a CBCT scan, information of the future prosthesis is added to the image accordingly. By incorporating standardized markers into the scan prosthesis, a “single-scan” protocol is performed [9]. The image with the scan prosthesis is registered with the stored data of the standardized marker in the software, and virtual implant planning related to the bony structure and prosthetic information can then be performed. However, a special positioning device is required in this protocol to communicate the virtually planned implant position to the surgical guide, and the surgical guide is fabricated in the laboratory by a dental technician instead of by using a CAD/CAM procedure [10].

In the mid-1990s, a “double-scan” protocol was proposed by researchers at the University of Leuven [11]. Customized fiducial markers made of gutta percha spheres were incorporated into the scan prosthesis. In addition to the CBCT scan with the patient wearing the scan prosthesis, the prosthesis alone was subjected to another CBCT scan. As the fiducial markers are visible in both sets of scans, the two scans can be registered by matching the fiducial markers, thus allowing implant planning with a 3D planning software. The surgical guide is then manufactured according to the digital image of the scan prosthesis. The disadvantages of the double-scan protocol include the extra time and cost required for fabricating the scan prosthesis. Moreover, if the intraoral seating of the scan prosthesis is not correct, the implant planning would be established on an invalid foundation, leading to incorrect implant positioning [6].

The introduction of the “image-fusion” procedure based on dental surface registration has excluded the need for a scan prosthesis in planning sCAIS [12,13]. A virtual dental model can be acquired by scanning the stone model or by using an intraoral scanner. By identifying some corresponding points on the dental surfaces of the digital dental cast and the 3D volume of the CBCT scan, these two images can be registered by matching the points with the best-fit algorithm, and the implant planning can proceed after merging the images. This procedure drastically reduced the time and cost of implant planning [12]. The possibility of human errors from laboratory fabrication and intraoral seating of the scan prosthesis can also be diminished. Moreover, the patient does not need a second CT scan with the scan prosthesis in place. Therefore, additional radiation exposure is avoided. Nonetheless, the dental surface registration method cannot be applied in certain clinical situations, for example, edentulous jaws or patients with only a few remaining teeth [14].

The accuracy of dental surface registration has been verified [14,15]. Moreover, sCAIS accuracy planned through dental surface registration has also been examined. Widmann et al. reported excellent in vitro accuracy of image-fusion SLA guides [12]. In a retrospective study of 12 patients, Schnutenhaus et al. reported a sufficiently high degree of accuracy of sCAIS without using the CBCT scan template [16]. However, to the best of our knowledge, no study has compared the in vivo accuracy between sCAIS planned through dental surface registration and fiducial marker registration. The present study therefore examined and compared sCAIS accuracy planning with dental surface registration and fiducial marker registration. The null hypothesis was that sCAIS planned through dental surface registration is as accurate as that planned through fiducial marker registration.

## 2. Materials and Methods

### 2.1. Patient Enrollment

This clinical trial was approved by the Institutional Review Board of Shin Kong Wu Ho-Su Memorial Hospital, Taipei, Taiwan (trial no. 20141004R), and was executed between 1 January 2015, and 31 December 2015. The inclusion criteria were as follows: patients with partial edentulism with healthy periodontal condition of the remaining teeth or fully edentulous patients who were willing to receive dental implants. Patients who had major systemic diseases, were under bisphosphonate therapy, or were pregnant were excluded. Patients with the following oral conditions were also excluded: a large amount of bone grafting was necessary for the implant site, and mouth opening was not wide enough to perform the sCAIS. A total of 30 patients were enrolled in this study. All patients signed the informed consent form and received a thorough oral and radiographic examination before the study.

### 2.2. Matching the Digital Dental Cast with the CBCT Scan

**Figure 1:**
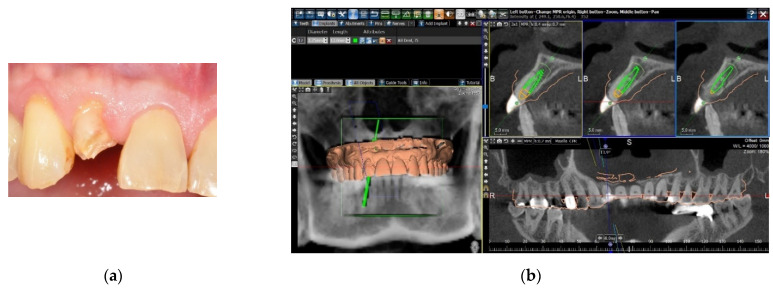
Matching the digital dental cast and the CBCT scan by registration of dental surface images. (**a**) A patient with a fractured right maxillary lateral incisor. (**b**) The digital dental cast and the CBCT scan were superimposed by setting registration points on the corresponding dental surfaces of the two digital images.

Two methods were used for the image registration of the digital dental cast and the dataset of the CBCT scan. The first was to take an impression of the patient’s dental arch with polyether impression material (Impregum Penta Soft, 3M Deutschland GmbH, Neuss, Germany), and a stone cast was poured. The stone cast was optically scanned with a desktop model scanner (inEos X5, Sirona Dental Systems GmbH, Bensheim, Germany) to obtain a digital dental cast in the form of Standard Tessellation Language (STL) files. A CBCT scan of the patient was taken with a large field-of-view CBCT scanner (3D eXam, KaVo Dental, Biberach, Germany), and the Digital Imaging and Communication in Medicine (DICOM) files were attained. The two data sets were uploaded into implant planning software (BenQ AB Guided Service, AB Dental, Ashdod, Israel). The two digital images were then superimposed with the registration tool of the software by setting five registration points on the corresponding dental surfaces of the two digital images (Figure 1).

**Figure 2:**
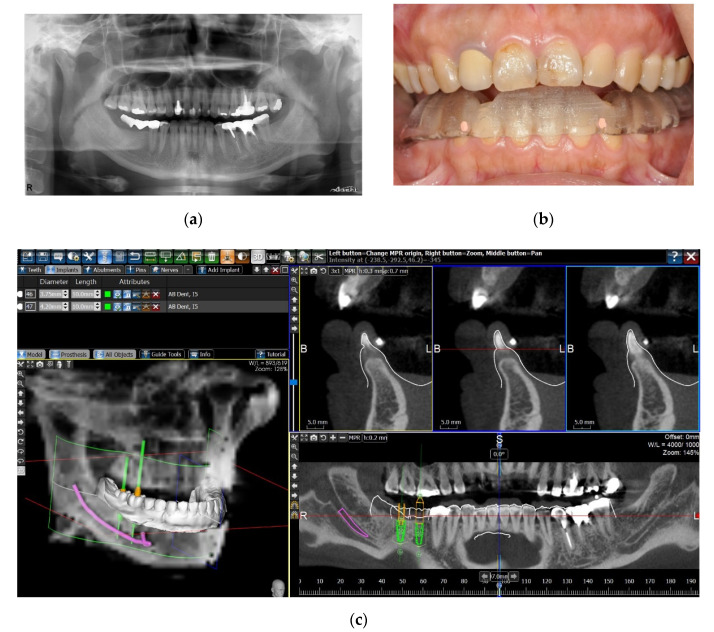
Matching the digital dental cast and the CBCT scan by registration of fiducial markers. (**a**) A patient with missing right mandibular first and second molars. Because of the streaking artifacts from the radiopaque crowns of bilateral posterior area on the CBCT scan, a direct superimposition with the digital dental cast was challenging. (**b**) On the basis of the digital dental cast, an SLA radiographic template with gutta percha fiducial markers was fabricated. The patient took a CBCT scan with the radiographic template in the mouth. (**c**) The image of the fiducial markers could be identified on both the digital dental cast and the CBCT scan, and the two digital images could be superimposed by matching the fiducial markers.

In some cases, insufficient registration points could be set on the dental surface of the digital cast; for example, the number of remaining teeth was not sufficient, the remaining teeth were not satisfactorily distributed for a precise registration, the patients were edentulous, or the streaking artifacts of radiopaque restorations on the CBCT scan hindered the correct registration process. To overcome this challenge, a second method of image registration was chosen. A radiographic template was first designed and fabricated by the SLA process based on the digital dental cast. Radiopaque fiducial markers were set on the template, and the patient underwent a CBCT scan with the SLA radiographic template in the mouth. The image of the fiducial markers could then be identified on both the digital dental cast and the CBCT scan, and the two digital images could be superimposed with the same registration tool of the software, using the fiducial markers as the registration points (Figure 2).

### 2.3. Guided Implant Surgery and Measurement of Deviations

The following materials and procedures have been described in an earlier report [17]. In short, one experienced technician verified the registration of the digital dental cast and the CBCT scan. Virtual implant planning was made considering the anatomic and restorative situations, and accordingly an SLA surgical guide (BenQ AB Guide, BenQ AB DentCare, Taipei, Taiwan) was fabricated by a certificated manufacturer. Standard metal sleeves specific to the guided surgery system (4.5 mm in height and 5.0 mm in diameter) were set into the surgical guide. For some patients with a limited mouth opening, the metal sleeve was designed to be C-shaped with an opening on the buccal side to facilitate side-entry of the surgical drills.

**Figure 3:**
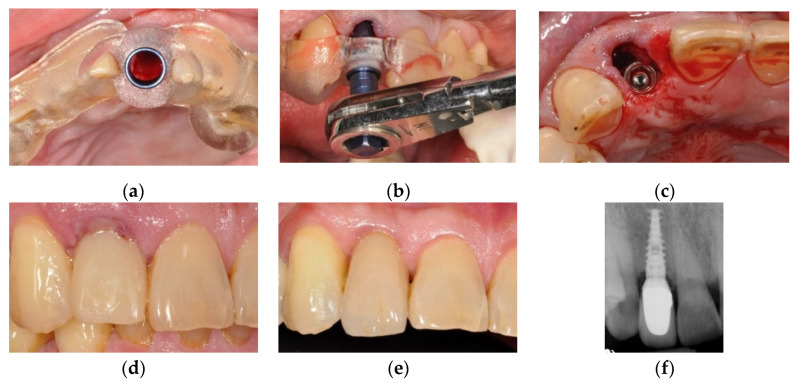
Clinical procedures of the patient in Figure 1. (**a**) The surgical guide was checked for correct seating. (**b**) Fully guided implant placement through the surgical guide. (**c**) The implant was inserted at the planned position. (**d**) Provisional crown in place the subsequent day after surgery. (**e**) Finished crown restoration after 6 months of healing. (**f**) Radiograph of the finished restoration.

One author (CC Lin) performed all the planning and surgeries of the sCAIS. After the stability and the position of the surgical guide were checked in the patient’s mouth, the sequential drilling procedures were executed using the BenQ AB Guided Service System (BenQ AB DentCare, Taipei, Taiwan) and bone-level type implants (I5 Conical Implant, AB dental, Ashdod, Israel). Fully guided implant placement was attempted whenever possible. Figure 3 illustrates the simplified clinical procedures of the guided surgery.

Right after the implant insertion, the patient underwent a postoperative CBCT scan to obtain the placed implant position; this was approved by the ethical committee with the informed consent of the patients. Using the same registration tool of the above-mentioned implant planning software, the pre- and postoperative CBCT images of the jaw were matched. The image of the actually placed implant could be subsequently identified, and then, the deviation between the planned and placed implants could be measured with metrology software (Geomagic Control X, 3D Systems Inc., Rock Hill, SC, USA).

**Figure 4:**
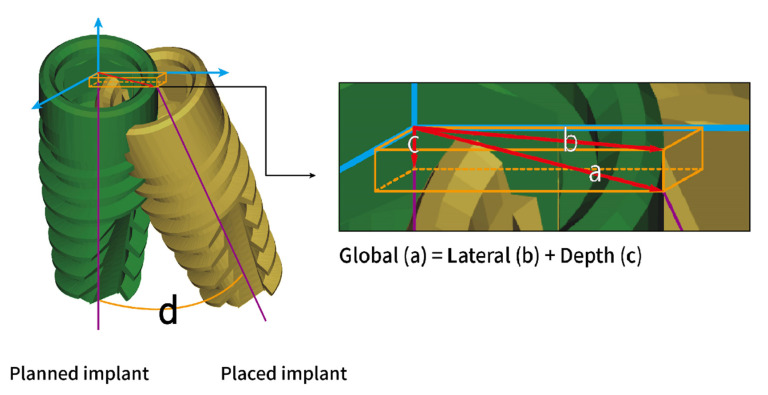
On the basis of the coordinate system created according to the planned implant position (blue arrows), the following spatial deviations (red arrows) and angular deviation between planned and placed implants were defined and measured: (**a**) global deviation, (**b**) lateral deviation, (**c**) depth deviation, and (**d**) angular deviation.

**Figure 5:**
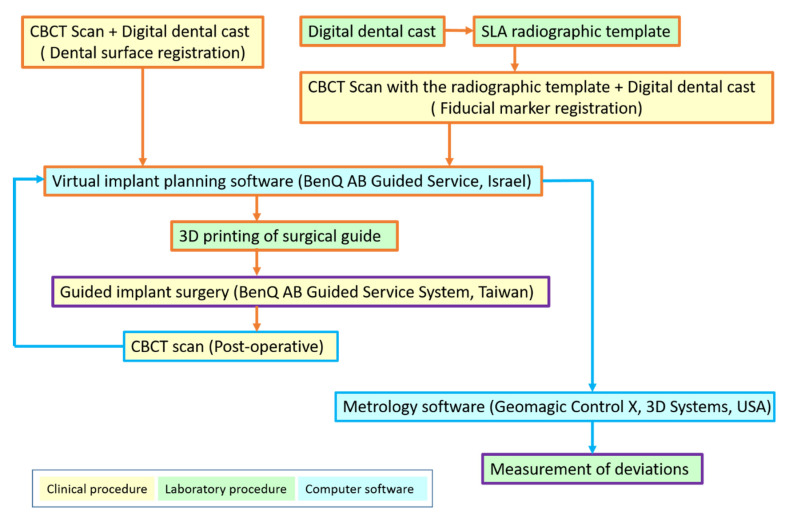
Flowchart summarizing the procedures of this study. Orange arrows indicate the planning and execution of the guided surgery, and blue arrows indicate the deviation measurement.

The subsequent spatial and angular deviations of planned and placed implants were measured: the global/lateral deviations at the implant platform, global/lateral deviations at the implant apex, depth deviation, and angular deviation. Global deviation is defined as the three-dimensional distance between the center of the implant platform/apex. The lateral deviation is the distance between the implant centers at the level of the planned implant platform/apex. The depth deviation is the distance of the centers of the implant platform at the axis of the planned implant. The angular deviation is the three-dimensional angle between the implant axis (Figure 4). A simplified flowchart summarizing the procedures of this study is illustrated in Figure 5.

### 2.4. Statistical Analysis

The sample size of 30 was calculated from a pilot study of simulation on the dental model, with a 0.5 effect size, 80% power, and 5%α-error. The outcome variables were deviation measurements between the placed and planned implants, including global/lateral deviations at the implant platform, global/lateral deviations at the implant apex, depth deviation, and angular deviation. The primary independent variable was the image registration method of the digital dental cast and the CBCT scan (by dental surface registration or by fiducial marker registration). Other clinical variables that could influence the accuracy were also classified as follows: implant site (incisor/canine, premolar, molar), jaw position (maxilla or mandible), implant site bone quality (types I, II, III, IV), implant length (8/10 mm, 11.5 mm, 13/16 mm), implant diameter (3.5 mm, 3.75 mm, 4.2 mm), the surgical technique (open flap or flapless surgery), the timing of implant placement (immediate or early/delayed implant placement), the surgical guide support (mucosa-supported, bilaterally tooth-supported or one-side tooth-supported in distal extension situation), and the type of metal sleeve in the surgical guide (standard or side-entry).

Variables are presented as mean ± standard deviation and range. Box plots were used to demonstrate the distribution of deviations. Intergroup differences were examined with a two-sample *t* test and one-way analysis of variance (ANOVA); for multiple comparison analysis, Tukey’s method was used in ANOVA. The multiway ANOVA was used to adjust the interaction between independent variables if more than one significant independent variable was identified. STATA 14 (StataCorp, College Station, TX, USA) was used for all statistical analyses. A *p* value of <0.05 was considered significant.

## 3. Results

The mean age of the 30 patients (18 men and 12 women) was 56.0 years (range: 24–80 years). With regard to the planning procedure of matching the digital dental cast and CBCT image, 22 patients were based on dental surface registration, and 8 were based on fiducial marker registration (4 partially edentulous and 4 fully edentulous patients). A total of 33 sCAIS were performed, in which three patients received two implant surgeries at different sites. The surgical sites consisted of 15 single tooth gaps, 12 partially edentulous ridges with over two teeth missing, and six edentulous jaws. A descriptive analysis of the patients and their guided surgeries is presented in Table 1.

A total of 74 implants were placed. However, 18 had osteotomy procedures with the help of the surgical guide but had to be placed free-handed (partially guided). The following situations accounted for free-handed implant insertion: the limited mouth opening of the patient in the molar site, limited space between adjacent teeth precluding the placement of a metal sleeve on the surgical guide, or large diameter of the implant prohibiting the guided insertion through the sleeve. Therefore, only 56 implants were inserted fully guided through the SLA surgical guide.

The comparison between fully guided and partially guided implants, together with the data of all 74 implants in this trial, is presented in Table 2. For the 56 fully guided implants, the mean global deviations were 0.97 ± 0.45 mm at implant platform and 1.27 ± 0.58 mm at implant apex. The mean lateral deviations were 0.69 ± 0.41 at implant platform and 1.04 ± 0.58 mm at implant apex. The mean depth deviation was 0.57 ± 0.43 mm, and the mean angular deviation was 3.21 ± 1.72°. All the measured mean deviations of partially guided implants were significantly larger than those of fully guided implants.

**Figure 6:**
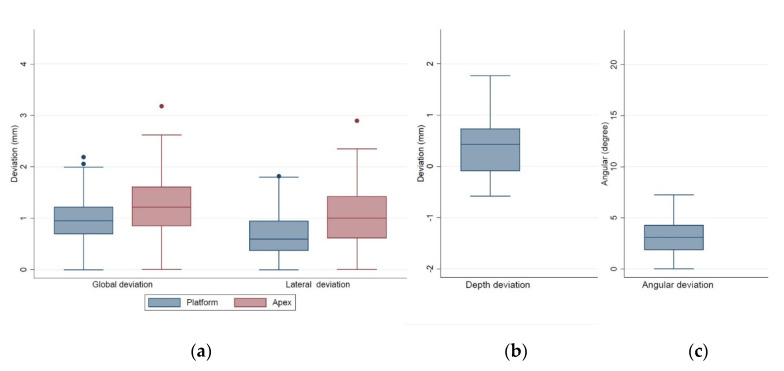
Box plots of the deviations of the 56 fully guided implants. (**a**) global/lateral deviations at implant platform and at implant apex, (**b**) depth deviation, and (**c**) angular deviation.

The distributions of deviations of the 56 fully guided implants are shown in the box plots in Figure 6. In general, the deviations at the implant platform were smaller than at the implant apex. At the implant platform, 50% of the values of the lateral deviation were <0.60 mm and 50% of the values of the global deviation were <0.94 mm (Figure 6a). Most implants were placed not considerably deeper than the predetermined position (the lower quartile was −0.08 mm, Figure 6b). Fifty percent of the angular deviations were smaller than 3.08°, and only 25% were larger than 4.26° (upper quartile, Figure 6c).

For the 56 fully guided implants, 3 variables were identified as having a significant influence on the deviation, and the comparison of deviations between inserted and planned implants by the 3 variables is presented in Table 3. Implants planned based on the dental surface registration indicated a significantly larger lateral deviation at the implant apex (1.24 ± 0.68 mm) than those based on the fiducial marker registration (0.92 ± 0.49 mm, *p* = 0.0464). Implants placed in the mandible had significantly lower lateral deviation at the implant platform (0.54 ± 0.29 mm) than in the maxilla (0.90 ± 0.47 mm, *p* = 0.0023). Significant differences were observed between implants placed through mucosa-supported surgical guides and those placed through unilaterally tooth-supported surgical guides in distal extension situations, for the lateral deviation at the implant platform (0.62 ± 0.33 mm and 1.03 ± 0.61 mm, respectively, *p* = 0.0321) and at the implant apex (0.88 ± 0.49 mm and 1.55 ± 0.68 mm, respectively, *p* = 0.0127). However, no significant differences were observed between the groups using bilaterally tooth-supported surgical guides and unilaterally tooth-supported surgical guides in distal extension situations, although the distal extension group had larger mean values of positional and angular deviations. For the other variables, no significant differences were found for the analyzed spatial and angular deviations.

Table 4 demonstrates the three-way ANOVA results that considered the mutual influence and the possible interaction of the three significant independent variables identified by the one-way ANOVA. After adjustment for the effects of the other two significant factors and their interactions, significant differences were found between the dental surface registration method and the fiducial marker registration method on the lateral deviation at both implant platform and apex (*p* = 0.0188 and 0.0371, respectively). The jaw position had a significant influence on the lateral deviation at the implant platform (*p* = 0.0002). For surgical guide support, the distal extension group exhibited significantly larger lateral deviation at the implant platform than the bilaterally tooth-supported group and the mucosa-supported group (*p* = 0.01). Moreover, the distal extension group showed significantly larger lateral deviation at the implant apex than the mucosa-supported group (*p* = 0.0117).

## 4. Discussion

The expanding development of digital technology has led to the alteration of the digital workflow of sCAIS. For implant planning, the point-based, dental surface registration method has been adopted for almost one decade and is extensively used nowadays [18]. Although favorable accuracy was demonstrated for the dental surface registration and for the sCAIS planned through dental surface registration in the literature [12,14,15,16], the results of the present study showed that, after adjustment for other potential influencing factors, significantly larger lateral deviations at both the implant platform and apex (*p* = 0.0188 and 0.0371, respectively) were found for the sCAIS planned through the dental surface registration method compared with that planned through the fiducial marker registration method. The null hypothesis was rejected.

The digital workflow of the sCAIS consists of data acquisition and processing, prosthetic-implant planning, surgical guide production, and execution of the guided implant surgery. Because the surgical guide is generated according to the data of the digital dental cast, to visualize the alveolar bone and other vital anatomic structures together with the digital dental cast, the CBCT dataset has to be matched to the digital dental cast before appropriate implant planning can proceed. Undoubtedly, this registration procedure is the most critical step in data processing. It establishes the basis of the entire digital workflow of the sCAIS. If the digital cast was not correctly matched to the bony image, the actual implant position would not be the same as that planned in the software. Any mistakes in this step will lead to consequent errors and the final inaccuracy of the implant placement [19].

The point-based dental surface registration method is cost-efficient and time-saving because the radiographic scan template is no longer necessary for patients with more than five residual teeth [14]. Normally, this image registration process uses a best-fit algorithm of the implant planning software. After at least three corresponding points on each image are selected by the operator, the two images can be merged automatically using software. The operator can check the approximation of the two images and make adjustments manually, if necessary, to improve the registration. Kim et al. [20] examined the accuracy of integration between digital dental casts and three-dimensional CT images by point-based markerless registration. An average error of 0–0.2 mm was measured, and they concluded that for the registration of dental models and maxillofacial CT images, a high accuracy can be achieved without the help of fiducial markers. Schnutenhaus et al. [14] compared the accuracy of the match with and without a radiographic template between CBCT and model scan data. They observed a 0.2-mm matching accuracy and interpreted that because the resolution of CBCT was 0.2 voxel, a matching accuracy of <0.2 mm was not possible. Therefore, the accuracy of matching without reference markers is adequate for planning the sCAIS.

However, the in vivo outcome of the present study did not coincide with the above-mentioned laboratory research. Several variables could affect the outcome of dental surface registration. The accuracy may vary depending on the computer software programs [18]. The operator of the registration process also plays a vital role in the outcome. This procedure is very subjective and depends on the skill of the operator [21]. Since matching point selection mainly relies on the operator’s visual observations, registration accuracy may be affected by human error in matching point selection [18]. Critically, the original image quality of CBCT could profoundly affect the result of the best-fit algorithm process. Indistinctive radiographic images can lead to a poor result of the registration [22]. Flügge et al. used CBCT data and intraoral surface scans of 36 patients to examine the influence of the operator, the preprocessing of data, and image artifacts on registration accuracy [19]. They concluded that the registration accuracy is significantly influenced by the operator, the segmentation processing of CBCT data, and the number of restorations in the patient’s mouth. Registration inaccuracy increased significantly with the number of restorations. In an in vitro study, Kim et al. [23] investigated the influence of metal artifacts within CBCT data on sCAIS accuracy and concluded that the presence of metal restorations resulted in significantly larger deviations at both the implant platform and apex.

In the present study, the same CBCT device and resolution were used for all the patients. Patients were carefully monitored during the radiographic procedure to avoid movement artifacts. Therefore, the only variable regarding the quality of the CBCT image was the scattering artifact from the radiopaque restorations. Among the 22 patients planned through dental surface registration in this study, only 3 had no metal crowns, and over half of the patients (13) had more than three crown restorations in the studied jaw. Because all the CBCT segmentation and registration procedures were performed by one experienced technician and all the implant surgeries were performed by one doctor according to the same materials and protocol, the influence of the operator and the materials should be minimum in this study. After adjustment for the other clinical variables of the patients, we believed that the streaking artifact of metal restorations within CBCT data should be the most influential variable that affected the registration of CBCT and digital dental cast and the consequent accuracy of the sCAIS. The streaking artifacts normally extended from the crown portion of the tooth and projected out in the buccal–lingual direction. This phenomenon might cause difficulty in appropriately matching the buccal and lingual surfaces of the teeth, thus explaining why only the lateral deviations were affected.

In the present study, the mean lateral deviations for the surface registration method (0.83 ± 0.51 mm at implant platform and 1.24 ± 0.68 mm at implant apex) were comparable to other in vivo studies. Bover-Ramos et al. [24] performed a systematic review of 2244 implants from 22 in vivo studies and observed overall mean lateral deviations of 1.10 ± 0.09 mm/1.40 ± 0.12 mm at the implant platform/apex, a mean depth deviation of 0.74 ± 0.10 mm, and a mean angular deviation of 3.98 ± 0.33°. Using a similar dental surface registration method as that in this study for implant planning, Schnutenhaus et al. [16] examined 12 cases with single tooth gap or distal extension situations. They revealed a mean horizontal deviation of 0.9/1.0 mm at the implant neck, a mean horizontal deviation of 1.5/1.6 mm at the implant apex and a mean angular deviation of 4°/5° for single tooth gap/distal extension situations, respectively. Similarly, by matching scanned dental model and CBCT images for implant planning, Lee et al. [25] measured a mean lateral deviation of 1.09/1.56 mm at the implant neck/apex and a mean angular deviation of 3.80° from 102 implants in 48 patients. In the present study, the outcomes of the dental surface registration group (0.83/1.24 mm for mean lateral deviation at the implant platform/apex, 0.55 mm for mean depth deviation, and 3.13° for mean angular deviation) were satisfactory compared with other studies, thereby justifying the clinical use of the surface registration method for planning sCAIS.

Sufficient accuracy was also achieved for the fiducial marker registration method in this study (0.60/0.92 mm for mean lateral deviation at the implant platform/apex, 0.58 mm for mean depth deviation, and 3.26° for mean angular deviation). Various studies have examined the in vivo accuracy of the implant placement of the sCAIS conducted with the conventional double-scan protocol. The mean total error at the implant platform ranged from 0.7 to 1.7 mm [6]. Compared with the conventional double-scan protocol, the global deviations at the implant platform (0.92 mm) of the fiducial marker group in this study were comparable and satisfactory. Although the double-scan protocol and the fiducial marker registration method used in this study both use radiopaque markers as the reference points for the registration, one major difference between them was that, instead of being fabricated manually in the dental laboratory, the radiographic template was generated through SLA production in this study. For fiducial marker registration, the correct intraoral position of the radiographic template during CBCT scanning is crucial. Errors in positioning will lead to consequent errors in image registration and inaccuracy of the sCAIS. On the basis of the data of the digital dental cast, the SLA-generated radiographic template could be more precise and facilitate the correct intraoral seating. This could be another reason for the more favorable results observed in this study.

In a systematic review comparing the accuracy of different types of implant placement through meta-analysis, Gargallo-Albiol et al. [26] concluded that for sCAIS, the fully guided implant placement had the highest accuracy, followed by the half-guided placement, whereas the freehand implant placement had the least accuracy. Similar results for the fully guided vs. partially guided implant placement were reported in other systematic reviews [24,27]. The present study also demonstrated that fully guided placement was more accurate than partially guided placement. Except for the lateral deviation at the implant apex, all the *p* values for the measured deviations were smaller than 0.01. This indicates that inserting implants through the SLA surgical guides was significantly more accurate and should be recommended when the accuracy of implant placement is essential.

In the literature, several variables that could affect sCAIS accuracy have been proposed and investigated, including the location of the implant site [28], alveolar bone quality [28], implant length [23,28], implant diameter [29], geometry of the metal sleeve [30], and flap or flapless approach [8]. However, in the present study, none of these variables had a significant influence on sCAIS accuracy.

The jaw position of the implant site has been reported to influence sCAIS accuracy; however, this finding is controversial [6]. Several studies have observed no differences between the maxilla and the mandible [30,31], whereas others have reported a higher accuracy for the mandible [32,33]. However, Sun et al. used a double-scan procedure and reported that for edentulous cases, the depth and angular deviations were significantly smaller in the maxilla than in the mandible [34]. They believed that the larger supporting areas in the maxilla result in better stability of the surgical guide, and hence, higher accuracy of the placed implants. Unlike their study, both dentate and edentulous patients were enrolled in the present study, and implants inserted into the mandible were more accurate than those inserted into the maxilla in terms of the lateral deviation at the implant platform. Moreover, we used dental surface registration and fiducial marker registration instead of a double-scan procedure. High-contrast CBCT images are required for correct image registration. The denser mandibular bone facilitates the segmentation procedure of the CBCT dataset and subsequent image registration, which may explain the lower lateral deviation in the mandible. The higher mandibular bone density could also aid in the confinement of the guided drilling and subsequent implant placement. However, because the effect of jaw position on sCAIS accuracy was not consistent in the literature, the role of jaw position may not be clinically significant. More research is warranted to clarify this issue.

Tissue support plays a critical role in sCAIS accuracy [23,30]. No significant differences were reported regarding sCAIS accuracy between mucosa-supported surgical guides and tooth-supported surgical guides in the meta-analyses of two systematic reviews [35,36]. However, the location and the number of missing teeth should also be considered. In the present study, the differences between bilaterally and unilaterally tooth-supported surgical guides were further examined. The differences between mucosa-supported guides and bilaterally tooth-supported guides were not significant. Not unexpectedly, after statistical adjustment of the registration method and the jaw position of the implant site, significantly larger lateral deviations were observed for the unilaterally tooth-supported guide than for the bilaterally tooth-supported guide and the mucosa-supported guide. For a distal extension situation with posterior teeth missing, due to insufficient support at the free end, the one-side supported surgical guide could be tilting or bending during the osteotomy and implantation procedure. This explained the larger lateral deviations of the unilaterally tooth-supported group, and similar results were also reported previously [37]. Hence, care must be taken when applying one-side supported surgical guides, especially when the extension length is more than two teeth. More evidence is required to verify this issue due to the limited number (eight) of implants in the unilaterally tooth-supported group in this study.

The major limitation of this study was that the included patients had different numbers, sizes, and locations of restorations in the mouth; hence, our results may not be applicable to patients without metallic restorations. However, patients may have restorations in reality; therefore, this real-world situation should be considered whenever we use the dental surface registration technique for planning sCAIS. Future well-controlled studies should evaluate the clinical impact of the numbers or locations of radiopaque restorations on sCAIS accuracy planned through dental surface image restoration.

To date, the dental surface image restoration method can only be applied to dentate patients. The number of residual teeth and their distribution in the dental arch may also influence the outcome of dental surface registration. Future studies should elucidate this issue. Advancements in technology may widen the application of this efficient and cost-effective method to edentulous patients.

According to the EAO consensus about computer-guided implant treatment in 2012, a mean system error of 1.2 mm in a horizontal direction and 0.5 mm in the vertical direction should be considered [38]. In the present study, accuracy improved in both dental surface and fiducial marker registration groups. However, although relatively low average deviations of implant placement can be achieved with sCAIS nowadays, significant large deviations were observed in a few cases. Surgeons should always keep this in mind; we strongly recommend a 2-mm safety margin when planning and performing sCAIS.

## 5. Conclusions

Within the limits of this study, we concluded that significantly larger mean lateral deviations at both the implant platform and apex were found for the sCAIS planned through the dental surface registration than for that planned through the fiducial marker registration. Although the sCAIS planned through dental surface registration was not statistically as accurate as that planned through fiducial marker registration, its accuracy was satisfactory and acceptable for clinical use. Further research is necessary to elucidate the underlying causes of this issue.

## Figures and Tables

**Table 1:** Description of the patients and surgeries.

Registration Protocol	Subject Number	Gender		Mean Age	Surgery Number	Fully Edentulous		Partially Edentulous	
		Male	Female	(Range)		Maxilla	Mandible	Maxilla	Mandible
Dental surface	22	14	8	55.1 ± 14.1	23 ^a^	-	-	13	10
				(24–80)					
Fiducial marker	8	4	4	58.1 ± 15.1	10 ^b^	1	5	2	2
				(32–80)					
Total	30	18	12	56.0 ± 13.3	33	1	5	15	12
				(24–80)					

^a^ one patient had two surgeries. ^b^ two patients had two surgeries.

**Table 2:** Comparison of deviations between fully and partially guided implants.

Implant Placement	Implant Number	Implant Platform		Implant Apex		Depth Deviation (mm)	Angular Deviation (Degree)
		Global (mm)	Lateral (mm)	Global (mm)	Lateral (mm)		
Total	74	1.18 ± 0.62	0.79 ± 0.45	1.51 ± 0.80	1.17± 0.72	0.74 ± 0.64	4.00 ± 2.51
(range)		(0.00–3.13)	(0.00–1.82)	(0.00–3.54)	(0.00–3.32)	(0.00–2.90)	(0.01–14.00)
Fully guided	56	0.97 ± 0.45	0.69 ± 0.41	1.27 ± 0.58	1.04 ± 0.58	0.57 ± 0.43	3.21 ± 1.72
(range)		(0.00–2.19)	(0.00–1.82)	(0.00–3.18)	(0.00–2.90)	(0.00–1.77)	(0.01–7.25)
Partially guided	18	1.84 ± 0.64	1.12 ± 0.40	2.24 ± 0.97	1.57 ± 0.96	1.26 ± 0.90	6.44 ± 3.02
(range)		(0.92–3.13)	(0.41–1.68)	(0.65–3.54)	(0.34–3.32)	(0.03–2.90)	(2.09–14.00)
*p* Value		<0.0001 *	0.0002 *	0.0006 *	0.0401 *	0.005 *	0.0003 *

* *p* < 0.05, Student’s *t* test.

**Table 3:** Deviations of variables that significantly influence fully guided implants.

Variables	Implant Number	Implant Platform		Implant Apex		Depth Deviation (mm)	Angular Deviation (Degree)
		Global (mm)	Lateral (mm)	Global (mm)	Lateral (mm)		
Image registration							
Dental surface	21	1.05 ± 0.57	0.83 ± 0.51	1.41 ± 0.73	1.24 ± 0.68	0.55 ± 0.44	3.13 ± 1.93
Fiducial marker	35	0.92 ± 0.37	0.60 ± 0.32	1.19 ± 0.46	0.92 ± 0.49	0.58 ± 0.43	3.26 ± 1.62
*p* Value		0.3505	0.0795	0.2259	0.0464 *	0.7745	0.7843
Jaw position							
Maxilla	23	1.07 ± 0.54	0.90 ± 0.47	1.34 ± 0.77	1.18 ± 0.75	0.49 ± 0.41	3.67 ± 2.14
Mandible	33	0.90 ± 0.37	0.54 ± 0.29	1.22 ± 0.40	0.95 ± 0.41	0.62 ± 0.44	2.89 ± 1.30
*p* Value		0.1663	0.0023 *	0.5130	0.1889	0.2709	0.1306
Guide support							
Mucosa-supported	28	0.98 ± 0.37	0.62 ± 0.33 ^b^	1.18 ± 0.47	0.88 ± 0.49 ^b^	0.64 ± 0.44	3.12 ± 1.53
Bilateral tooth support	20	0.89 ± 0.45	0.65 ± 0.36	1.25 ± 0.62	1.08 ± 0.57	0.53 ± 0.40	3.04 ± 1.89
Distal extension	8	1.15 ± 0.68	1.03 ± 0.61 ^a^	1.63 ± 0.75	1.55 ± 0.68 ^a^	0.42 ± 0.42	3.97 ± 1.96
*p* Value		0.4047	0.0321 *	0.1536	0.0127 *	0.4013	0.4116

* *p* < 0.05, *p* Value: Student’s *t* test or ANOVA test, ^a,b^ significant difference by Tukey multiple comparisons.

**Table 4:** Three-way ANOVA results of the significant independent variables in Table 3.

Variables	Implant Platform		Implant Apex		Depth Deviation (mm)	Angular Deviation (Degree)
	Global (mm)	Lateral (mm)	Global (mm)	Lateral (mm)		
Image registration						
*F* Value	1.28	5.92	2.07	4.6	0.09	0.08
*p* Value	0.2638	0.0188 *	0.157	0.0371 *	0.7698	0.7843
Jaw position						
*F* Value	2.23	15.95	0.6	2.52	1.27	2.71
*p* Value	0.1417	0.0002 *	0.441	0.1189	0.2663	0.1062
Guide support						
*F* Value	1.06	5.08	2.07	4.88	0.96	0.89
*p* Value	0.3529	0.01 *^,‡^	0.1368	0.0117 *^,†^	0.3915	0.4166

* *p* < 0.05, ^‡^ Distal extension > Bilateral tooth support; Distal extension > Mucosa support, ^†^ Distal extension > Mucosa support.
